# Molecular Mechanisms, Endurance Athlete, and Synergistic Therapeutic Effects of Marine‐Derived Antioxidant Astaxanthin Supplementation and Exercise in Cancer, Metabolic Diseases, and Healthy Individuals

**DOI:** 10.1002/fsn3.70470

**Published:** 2025-06-27

**Authors:** Wenwen Nie, Jianmin Li, Sogand Rajabi

**Affiliations:** ^1^ Physical Education Institute Qilu Normal University Jinan, Shandong China; ^2^ School of Tai Chi Culture Handan University Handan, Hebei China; ^3^ Department of Cellular and Molecular Biology Islamic Azad University, Sirjan Branch Sirjan Iran

**Keywords:** astaxanthin, cancer, cognitive function, exercercise, molecular signaling

## Abstract

Marine‐derived antioxidant astaxanthin (AST), a potent antioxidant carotenoid, has gained significant attention for its potential therapeutic effects in various diseases, including cancer and metabolic disorders. When combined with exercise, which is a well‐established intervention for improving health, these two modalities may offer synergistic benefits that extend beyond their individual effects. This review explores the molecular mechanisms underlying the synergistic therapeutic effects of AST and exercise in cancer, metabolic diseases, and healthy individuals. AST exerts its beneficial effects primarily through its ability to reduce oxidative stress, modulate inflammation, and enhance cellular signaling pathways, including those involved in apoptosis, autophagy, and mitochondrial function. It has been shown to suppress tumor growth, improve insulin sensitivity, and protect against the adverse effects of chronic diseases, such as cardiovascular complications and neurodegenerative conditions. Similarly, exercise induces a wide array of molecular adaptations, including the activation of key metabolic pathways, enhancement of mitochondrial biogenesis, and modulation of inflammatory responses. These effects improve metabolic health, reduce cancer risk, and promote overall well‐being. The combination of AST supplementation and exercise may provide a more potent therapeutic strategy, targeting multiple molecular pathways simultaneously. This synergy may not only enhance the effectiveness of each intervention but also reduce the side effects commonly associated with pharmacological treatments. This review discussed the current evidence for the synergistic effects of AST and exercise, highlighted the molecular mechanisms involved, and suggested potential clinical applications for these interventions in cancer, metabolic diseases, and healthy populations.

## Graphical abstract

In metabolic disease, astaxanthin reduces lactate and carbohydrate (CHO) oxidation, while increasing fat oxidation, suggesting improved metabolic efficiency. In relation to exercise, astaxanthin enhances mitochondrial protection and biogenesis, boosts capillary blood flow, and improves oxygen delivery, all of which could lead to better endurance and recovery. In cancer, astaxanthin helps lower inflammation and oxidative stress, highlighting its potential role in cancer prevention or therapy support.

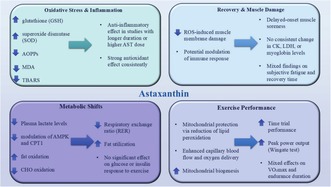

## 1 Introduction

Exercise has been used to prevent sarcopenia and noncommunicable illnesses, and to enhance sports performance. Enhanced muscular strength is one of resistance training's main adaptive effects. In general, the early stages of training are when neural impulse transmission and conduction are most active, which aids in the contraction of muscle fibers. Every muscle fiber eventually experiences hypertrophy as a result of consistent resistance training, which results in an even greater gain in strength. Energy expenditure is one of the skeletal muscle's primary functions. About 20% of the body's basal metabolism is made up of the enormous quantities of ATP that muscles make as they contract and utilize energy substrates (Gallagher et al. 1998; Bosy‐Westphal et al. 2004). As a result, when muscles get larger, the metabolic rate rises (Cunningham 1991; Nielsen et al. 2000). The body is constantly synthesizing new proteins and breaking them down. Muscle growth results from promoting protein synthesis and preventing protein catabolism. The best‐recognized method for promoting protein anabolism, which is mediated by several signaling pathways, is exercise. Moreover, during resistance training, proper dietary management speeds up gains in muscle development and strength. In general, skeletal muscle cells need to consume enough protein to provide amino acids as substrates for protein synthesis (Churchward‐Venne et al. 2012; Cholewa et al. 2017). By promoting insulin secretion, consuming proteins and carbs together can also effectively increase protein anabolism (Ivy et al. 2002). The beneficial effects of eating a macronutrient‐rich diet on training‐induced muscle adaptation have also been shown in several studies (Ivy et al. 2002; Berardi et al. 2006).

Sports science and nutrition are vast fields of study. Different exercise approaches and protocols such as intensity or time have been studied in terms of exercise training to investigate their unique impacts on the clinical outcomes of disorders including immunological response (Ismail et al. 2013), intraocular pressure (Conte et al. 2014), and cardiovascular illnesses (Ismail et al. 2013). Conversely, studies have examined the impact of various diets (Shannon et al. 2021; Pereira et al. 2023) and nutritional supplements (Bhatt and Patel 2020; Mussagy et al. 2019) as preventative measures or as ergogenic aids to improve exercise capacity (Jeukendrup 2017; Mujika et al. 2014). AST is one of the polyphenols among dietary supplements which is receiving consideration in the scientific works, particularly in the areas of aging, muscular recovery and fatigue, and, more recently, cognitive performance (Sztretye et al. 2019; Kim and Kim 2018; Higuera‐Ciapara et al. 2006). Notably, immunomodulatory effects have been demonstrated separately by AST (Higuera‐Ciapara et al. 2006) and exercise (Ismail et al. 2013), which is significant for the treatment and prevention of inflammatory complications like stroke, type 2 diabetes mellitus, and cancer. To ascertain how exercise and nutritional intervention might combine to have favorable impacts on health problems or diseases, concurrent AST ingestion and exercise training provide a viable method. Thus, with an emphasis on sports performance, molecular signaling, and cognitive function, this study attempted to gather and condense state‐of‐the‐art scientific knowledge about exercise training and AST supplementation.

## 2 Basis and Structure of AST

Although exercise and macronutrient‐rich diets are foundational for enhancing muscle performance and adaptation, recent attention has turned toward bioactive compounds that may further support these effects. Among these, AST, a naturally occurring carotenoid derived primarily from marine sources, has shown promising therapeutic potential due to its strong antioxidant and anti‐inflammatory properties. Understanding the biological basis and structural composition of AST is essential for appreciating its functional roles in human health and its synergistic effects when combined with exercise. This section outlines the natural origins, chemical structure, and bioavailable forms of AST, setting the stage for a deeper exploration of its mechanisms of action.

Algae, salmon, yeast, krill, trout, crayfish, and shrimp are the natural sources of AST. The primary sources of commercial AST are chemical synthesis, Haematococcus, and Phaffia yeast. One of the greatest natural AST sources is *Haematococcus pluvialis* (Ranga Rao et al. 2010; Ranga et al. 2009; Bampidis et al. 2023). The greatest AST level in wild Oncorhynchus species was found to be between 26 to 38 mg/kg meat in sockeye salmon, whereas chum had a low AST content (Bampidis et al. 2023). It has been found that the AST level of farmed Atlantic salmon is 6–8 mg/kg meat. Salmon that is wild‐fished is a potential source of AST. One may consume 165 g/day of salmon to obtain 3.6 mg of AST. A 3.6 mg daily AST supplement may be advantageous to health (Iwamoto et al. 2000). Because it has oxygen atoms in addition to carbon and hydrogen, AST is a member of the xanthophylls. A polyene chain connects the two terminal rings that makeup AST. This molecule has a hydroxyl group (–OH) on every end and 2 asymmetric carbons at the 3, 3′ locations of the β‐ionone ring. When a (–OH) binds with a fatty acid in the first instance, a mono‐ester is created; however, when two hydroxyl groups reply with fatty acids, a di‐ester is created. There are several forms of AST, including free, esterified, geometric, and stereoisomers (Ambati et al. 2014). In nature, the most prevalent stereoisomers are (3S, 3′S) and (3R, 3′R) (Hussein et al. 2006). Isomers of (3S, 3′S), (3R, 3′S), and (3R, 3′R) make up synthetic AST (Higuera‐Ciapara et al. 2006).

## 3 Antioxidant Effects of AST

**FIGURE 1:**
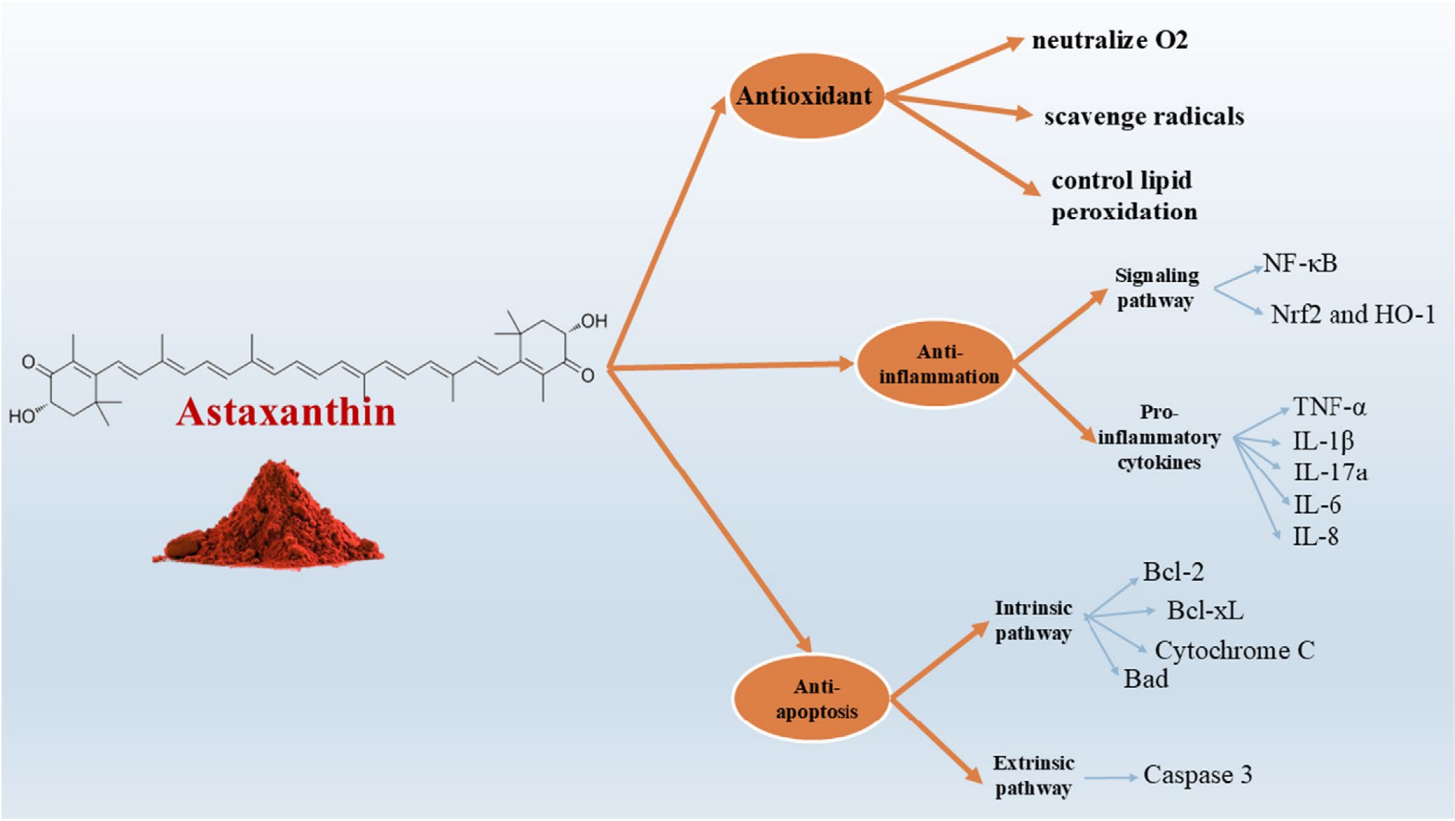
Mechanistic overview of astaxanthin (AST) supplementation effects on exercise performance, metabolic responses, oxidative stress, and recovery in human studies. Astaxanthin, a potent lipid‐soluble antioxidant, exerts diverse physiological effects relevant to exercise adaptation. It supports enhanced performance by protecting mitochondrial membranes from oxidative damage, promoting efficient oxygen transport, and potentially increasing mitochondrial biogenesis. AST may shift substrate utilization toward greater fat oxidation while lowering lactate accumulation during submaximal exercise, although results vary across studies. It consistently reduces oxidative stress by scavenging free radicals and upregulating endogenous antioxidant systems such as superoxide dismutase and glutathione. Anti‐inflammatory actions—possibly mediated via NF‐κB pathway inhibition—have been observed in longer‐duration interventions. Although AST may aid muscle recovery by reducing delayed‐onset muscle soreness (DOMS), its effect on classical muscle damage biomarkers (e.g., CK, LDH) remains inconclusive. The variability in human trial outcomes highlights the need for standardized protocols regarding AST dosing, duration, and subject training status.

AST's unique molecular structure not only defines its classification as a xanthophyll carotenoid but also underlies its powerful antioxidant capabilities (Figure 1). These antioxidant properties are central to its biological activity and therapeutic potential. Oxidative stress, caused by an imbalance between reactive oxygen species (ROS) and the body's ability to neutralize them, is a key contributor to cellular damage and the development of chronic diseases. In this section, we delve into the mechanisms by which AST mitigates oxidative damage, highlighting its role in neutralizing ROS, modulating redox‐sensitive signaling pathways, and preserving cellular integrity in various physiological and pathological contexts. In biological processes, molecular oxygen (O_2_) is the most important radical. ROS such as O_2_ are created by several physical and metabolic processes. Oxidative stress (OS) can be brought on by an excess of ROS (Hammarlund et al. 2020). Membranes, DNA, lipids, proteins, and other cell structures can all be negatively impacted by ROS and OS (Hammarlund et al. 2020; Jindrich and Degnan 2016). Owing to its special qualities, AST may neutralize O_2_, scavenge radicals, control lipid peroxidation (LPO), preserve immune system activity, the integrity of the cell membrane, and gene expression (Kamath et al. 2008; Vasanthkumar et al. 2010). Furthermore, some research suggests that AST has stronger antioxidant action than other carotenoids (Naguib 2000). Phosphoinositide 3‐kinase (PI3K)/protein kinase B (Akt) and Mitogen‐activated protein kinase (MAPK)/extracellular signal‐regulated protein kinase (ERK) pathways are both triggered by AST (Zarneshan et al. 2020). Nrf2 and Kelch‐like ECH‐associated protein 1 may be dissociated more easily because of these two routes. When Nrf2 joins the nucleus, the Nrf2 antioxidant response element (ARE) signaling pathway is triggered (Zarneshan et al. 2020). Both in vivo and in vitro protection against OS is provided by glutathione‐S‐transferase‐α1, the glutamate‐cysteine ligase modifier subunit, HO‐1, NAD(P)H quinone oxidoreductase‐1, and the glutamate‐cysteine ligase catalytic subunit, which are all upregulated by the PI3K/Akt pathway (Wu et al. 2015; Wang et al. 2010). AST has been shown to activate the PI3K/Akt and MAPK/ERK signaling pathways, both of which play complex roles in cellular growth, survival, and metabolism. Although these pathways are indeed implicated in the development and progression of certain cancers such as melanoma, AST appears to modulate them in a highly context‐dependent manner. In normal cells, AST‐induced activation of these pathways contributes to enhanced antioxidant defense, mitochondrial function, and cellular repair. However, in cancer cells, AST has been shown to exert antiproliferative and pro‐apoptotic effects, often through selective modulation of the same pathways, suggesting a regulatory rather than purely stimulatory influence. Importantly, existing preclinical and clinical studies have not reported tumorigenic effects associated with AST supplementation. On the contrary, AST has demonstrated anticancer potential through suppression of oxidative stress, inhibition of NF‐κB activity, and induction of apoptosis in various tumor models. Nevertheless, due caution and further research are warranted when considering AST use in oncology, especially in conjunction with conventional therapies (Figure 1).

## 4 Anti‐Inflammatory Effects of AST

In addition to its potent antioxidant activity, AST also exhibits significant anti‐inflammatory properties (Figure 1), which further contribute to its therapeutic value. Chronic inflammation is a hallmark of numerous diseases, including cancer, cardiovascular disorders, and metabolic syndromes, often acting in tandem with oxidative stress to drive disease progression. AST's ability to regulate key inflammatory pathways makes it a promising candidate for both disease prevention and recovery support in active individuals. This section explores the molecular mechanisms through which AST modulates inflammatory responses, with a focus on its interaction with the NF‐κB signaling pathway and the suppression of pro‐inflammatory cytokines (Netea et al. 2017; Monti et al. 2015). One of the main mechanisms by which AST affects inflammation is through regulating the NF‐κB signaling pathway. The nuclear transcription pathway known as the NF‐κB pathway is widely distributed and shows a crucial function in both immunological and inflammatory responses (Taniguchi and Karin 2018). Overstimulation of the NF‐κB signaling is linked to inflammatory changes in heart and brain disorders, as well as rheumatoid arthritis. When left unstimulated, NF‐κB stays dormant in the cytoplasm and engages in interactions with members of the repressing (IκB) family, namely IκB‐α (Ghosh et al. 1998). When extracellular substances stimulate IκB, they dissociate it and phosphorylate it through the IκB kinase complex (IKK) which contains IKKβ and IKKα. This process activates NF‐κB. Dissociated NF‐κB reaches the nucleus where it combines with κB regulatory elements to generate IL‐1β, tumor necrosis factor‐alpha (TNF‐α), and IL‐6, which are pro‐inflammatory markers (Liu et al. 2017; Almowallad et al. 2022). In a rat model of lung injury, it was shown that AST decreases NF‐κB as well as IL‐1β and TNF‐α and thereby decreases inflammatory response in these cells (Ozkanlar et al. 2025). This effect is approved by a great body of evidence (Ozkanlar et al. 2025; Sarker et al. 2024; Liu et al. 2025; Shafie et al. 2024; Jiang et al. 2024). In another study about the pathophysiology of cardiac hypertrophy and remodeling, it was detected that AST significantly reduced the inflammatory response by reducing the elevated levels of IL‐1β, IL‐17a, and TNF‐α (Sarker et al. 2024). Keap1‐Nrf2/HO‐1 signaling is another pathway by which AST is inhibiting inflammation. For instance, in a study on Dry eye disease (DED), Liu et al. (Liu et al. 2025) used immunofluorescence staining analyses and revealed a reduction in Keap1 protein levels in the corneal tissues of mice treated with AST, along with a notable increase in the proteins Nrf2 and HO‐1. Additionally, their in vitro experiments showed that AST significantly improved cell viability and decreased inflammation thereby providing protection to the human corneal epithelium (Liu et al. 2025). A clinical trial on Poor ovarian response (POR) patients also showed that (Shafie et al. 2024) The AST group exhibited significant decreases in serum levels of IL‐6 (*p* < 0.001), IL‐8 (*p* = 0.001), and VEGF (*p* = 0.002) after undergoing AST therapy. Additionally, in the AST group, the levels of IL‐6 (*p* < 0.001), IL‐8 (*p* = 0.036), VEGF (*p* = 0.006), and cfDNA (*p* < 0.001) in the follicular fluid (FF) were markedly lower compared to the placebo group (Shafie et al. 2024).

In a study on chronic prostatitis/chronic pelvic pain syndrome, Jiang and colleagues (Jiang et al. 2024) showed that oral administration of AST for 4 weeks in rats results in AST inhibiting the expression of proinflammatory cytokines such as interleukin‐1β (IL‐1β), IL‐6, IL‐8, and tumor necrosis factor‐α (TNF‐α). Additionally, AST reduced the activities of prostaglandin E2 (PGE2) and cyclooxygenase 2 (COX2). Moreover, AST diminished the activation of the nuclear factor‐κB (NF‐κB) signaling pathway (Jiang et al. 2024). Collectively, these findings highlight AST's multi‐pathway regulatory capacity, underscoring its potential as a therapeutic agent in inflammation‐related conditions. AST exerts powerful anti‐inflammatory effects by targeting several key molecular pathways involved in immune and inflammatory responses. One of its primary mechanisms is the inhibition of the NF‐κB signaling pathway, which regulates the expression of pro‐inflammatory cytokines such as IL‐1β, IL‐6, IL‐8, TNF‐α, and IL‐17a. Studies in various disease models—including lung injury, cardiac hypertrophy, and chronic pelvic pain—have consistently shown that AST reduces NF‐κB activation and downstream inflammatory mediators. Additionally, AST modulates the Keap1‐Nrf2/HO‐1 pathway, as evidenced by increased expression of Nrf2 and HO‐1 and decreased Keap1 in models of dry eye disease, leading to improved cellular resilience and reduced inflammation. Clinical evidence further supports AST's anti‐inflammatory role; for example, in patients with poor ovarian response, AST significantly lowered serum and FF levels of IL‐6, IL‐8, VEGF, and cfDNA.

## 5 Antiapoptotic Effects of AST

Beyond its antioxidant and anti‐inflammatory actions, AST also plays a crucial role in regulating cell survival through its antiapoptotic effects (Figure 1). Apoptosis, or programmed cell death, is essential for maintaining cellular homeostasis, yet excessive or dysregulated apoptosis is implicated in a range of pathological conditions, including neurodegenerative diseases and tissue damage induced by intense physical stress. AST has been shown to influence key molecular players in apoptotic signaling, helping preserve cell integrity and function. This section examines how AST interacts with apoptotic pathways, particularly through the modulation of Bcl‐2 family proteins and mitochondrial signaling cascades, to exert protective effects in various biological contexts (Grilo and Mantalaris 2019; Figueroa Jr. et al. 2007; Adams and Cory 2018). When apoptosis is stimulated, Bax and Bad accelerate the statement of cytochrome c (Cyt c) into the cytoplasm from the mitochondria. Apoptotic protease activator‐1, caspase‐9, and Cyt c form a complex that subsequently activates caspase‐3, instigating apoptosis. Apoptosis is induced and Cyt c release is suppressed by Bcl‐xL and Bcl‐2 (Czabotar et al. 2014; Chan and Yu 2004). Furthermore, the SCR/STAT or JAK/STAT pathways stimulate the production of Bcl‐xL and Bcl‐2, which contributes to antiapoptosis, whereas the PI3K/Akt route suppresses Bad and Bax. Apoptotic proteins that are important for disease prevention can be regulated by AST (Zhang and Wang 2015). Furthermore, researchers have demonstrated that AST plays a critical function in the PI3K/Akt pathway's activation, the modulation of Bad's phosphorylation, and the reduced expression of Cyt C and caspase‐3's activation (Dong et al. 2013; Zhang et al. 2014).

## 6 The Effects of AST on Sports Performance and Molecular Signaling Pathways in Animal Studies

**FIGURE 2:**
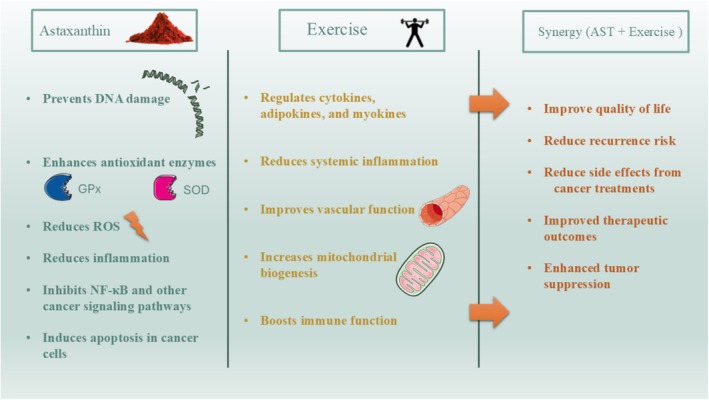
Synergistic anticancer mechanisms of astaxanthin (AST) and exercise. This figure illustrates the complementary biological pathways through which AST, a potent natural antioxidant derived from microalgae, and exercise exert anticancer effects. AST reduces oxidative stress, inflammation, and DNA damage by enhancing antioxidant enzyme activity and modulating key signaling pathways such as NF‐κB. Meanwhile, exercise improves immune function, increases mitochondrial biogenesis, and regulates systemic factors like cytokines and myokines. When combined, AST and exercise may act synergistically to enhance cancer cell apoptosis, suppress tumor growth, and improve overall therapeutic outcomes and quality of life. This integrated approach highlights the potential of combining nutritional and lifestyle interventions in cancer prevention and management.

Among conducted works on animal studies, there are 11 model studies correlated with the objective of this review (Table 1 and Figure 2). The impact of AST on muscle lipid metabolism during exercise was examined in a study on ICR mice (ICR (CD‐1) outbred mice, originally derived from Swiss albino mice). Following a 4‐week course of therapy, the exercise groups ran on treadmills. When AST‐fed mice were compared to others, they used more fat during activity, extending their running time till exhaustion. AST caused an increase in the carnitine palmitoyltransferase I (CPT I) and colocalization of fatty acid translocase in skeletal muscle. Additionally, it was shown that exercise enhanced the hexanoyl‐lysine change of CPT I, whereas AST inhibited this rise. Additionally, the decrease in body fat formation following exercise training was expedited by AST therapy (Aoi et al. 2008). The findings demonstrated that AST supplementation led to increased expression of CPT I and enhanced colocalization of fatty acid translocase in skeletal muscle, resulting in greater fat utilization during exercise. This shift toward lipid metabolism is beneficial as it supports prolonged energy supply during endurance activities, delays fatigue, and contributes to improved performance outcomes. Additionally, promoting fat oxidation over carbohydrate reliance may offer therapeutic advantages for individuals with metabolic disorders, such as obesity or type 2 diabetes, where improving lipid metabolism is a key target. These results underscore AST's role in enhancing energy efficiency and metabolic flexibility during physical exertion.

**TABLE 1:** The impacts of astaxanthin on molecular signaling and performance in animal studies.

Animal	Dosage	Duration	Primary outcome	Results	References
ICR mice	0.02% w/w	4 weeks	Muscle lipid metabolism	Improved lipid metabolism rather than glucose utilization	Aoi et al. (2008)
Training horses	75 mg/day	8 weeks	Exercise‐induced muscle damage	Attenuated exercise‐induced muscle damage	Sato et al. (2015)
Male C57BL/6 mice	5, 15, 30 mg/kg BW	4 weeks	Antioxidant enzyme activity	Enhancing antioxidant enzymes activity Downregulating Nrf2‐dependent enzymes And Nrf2 Attenuating muscle and plasma MDA	Zhou et al. (2019)
Wistar rats	1 mg/kg BW	45 days	Physical exhaustion	Enhanced antioxidant answers	Polotow et al. (2014)
Wistar rats	20 mg/kg	6 weeks	Oxidative stress injury	Developed overtraining syndrome, oxidative stress, apoptosis, and functional/morphological damage of skeletal muscle	Niu et al. (2021)
Mice	30 mg/kg BW	4 weeks	Endurance performance	Increased lipid metabolism	Ikeuchi et al. (2006)
ICR mice	1 mg/kg BW	6 weeks	Lipid profile	Enhanced PGC‐1alpha in skeletal muscle	Liu et al. (2014)
Wistar rats	0.04% w/w	24 days	Muscle atrophy	Improved skeletal muscle atrophy	Shibaguchi et al. (2016)
ICR mice	30 mg/kg BW	5 weeks	Strength performance	Enhanced time to exhaustion	Aoi et al. (2018)
Mice	0.02% w/w	24 weeks	Insulin metabolism	Enhanced mitochondria biogenesis	Nishida et al. (2020)
Mice	0.5% w/w	4 weeks	Cognitive function	Enhanced spatial memory	Yook et al. (2016)

In training horses, the effects of l‐carnitine and AST dietary supplements on blood biomarkers and the clinical occurrence rate of exercise‐made muscle injury were investigated. During the trial, 63 sound Thoroughbred horses were divided into 2 groups and given the identical base food and exercise regimen. For 8 weeks, the supplement group took 75 mg/day AST. Following high‐intensity exercise training, blood samples were taken three days, eight weeks, and five weeks before the commencement of the supplementation. Although the supplement group did not exhibit any significant difference, the control group's creatine kinase activity at week eight was considerably higher than it was on day three. The supplement group's creatine kinase activity was much lower than the control group's after 8 weeks. Furthermore, in comparison to the control group, the LDH‐5 levels in the supplement group tended to be reduced. In comparison to the control group, the incidence rate of exercise‐made muscle injury was noticeably reduced in the AST group (Sato et al. 2015).

Another study looked at whether antioxidant enzyme activity during moderate‐intensity exercise. For four weeks, 45 min a day of moderate‐intensity swimming exercise were paired with AST (5, 15, and 30 mg/kg BW) in male C57BL/6 mice. The findings demonstrated that compared with the swimming control group, the mice given 15 and 30 mg/kg of AST had lower levels of glutathione peroxidase, creatine kinase, catalase, and malondialdehyde in their muscle or plasma. Furthermore, the gastrocnemius muscle erythroid 2p45 (NF‐E2)‐Nrf2 was downregulated by these two AST concentrations of 15 and 30 mg/kg BW. In the meantime, the AST‐treated groups also showed downregulation of Nrf2 and Nrf2‐dependent enzymes' messenger RNA in the mice's hearts. However, the mice treated with 15 or 30 mg/kg AST had elevated superoxide dismutase (SOD) activity and nitric oxidase synthase, compared with the swimming and sedentary control groups (Zhou et al. 2019). In another research, it was shown that giving Wistar rats a long‐term (1 mg AST/kg BW) supplement for 45 days considerably postponed the time to fatigue by 29% during a swimming test. In the plasma of exercising animals, AST supplementation reduced exercise‐induced iron overload and its associated pro‐oxidant effects while increasing scavenging and iron‐chelating capabilities. AST significantly raised the amount of glutathione in the soleus muscles during exercise, reduced oxidative stress, and postponed fatigue via inducing cytosolic glutathione peroxidase and mitochondrial Mn‐dependent SOD responses (Polotow et al. 2014).

Another investigation examined AST in controlling protein expression associated with the nuclear factor erythroid 2‐related factor 2 (Nr12) signaling. Forty Wistar rats were divided into 3 groups at random: AST plus high‐intensity exercise (AH1), high‐intensity exercise (HI), and control group (C). HI and AHI had increasing load treadmill training for six. Rats in groups C and HI received an identical volume of soybean oil throughout training, but rats in group AHI received AST (20 mg/kg, 5 mL/kg). The findings showed that in comparison to rats in group C, rats in group HI experienced inflammatory changes in their skeletal muscle; apoptosis, Bax, and MDA significantly increased; serum T/Cor significantly decreased; CK and LDH significantly increased; and skeletal muscle Bcl‐2, Bc1‐2/Bax, SOD, and Nrf2, HO‐1 significantly decreased. Rats in group AID showed lower levels of inflammatory cells in their skeletal muscles than those in group III. They also showed significant increases in serum T/Cor, significant decreases in CK and LDH, significant increases in skeletal muscle Bcl‐2, Bc1‐2/Bax, SOD, Nr12, and HO‐1, and significant decreases in apoptosis, Bax, and MDA (Niu et al. 2021). To find out how AST affected the endurance ability of 4‐week‐old male mice, a study was designed. For five weeks, mice with stomach intubation received either vehicle or AST (1.2, 6, 30 mg/kg BW) orally. The AST group's swimming time to fatigue increased significantly. The AST group's blood lactate value was noticeably lesser than that of the control group. Swimming exercise reduced plasma nonesterified fatty acid (NEFA) and glucose levels in the control group, but increased plasma NEFA and glucose considerably in the AST group. Additionally, AST dramatically reduced fat buildup (Ikeuchi et al. 2006).

Liu et al. (2014), the impact of AST consumption on lipid metabolism in mice was examined with peroxisome proliferator‐activated receptor‐γ coactivator‐1α (PGC‐1α). Following a two‐week course of therapy, the exercise groups ran for thirty minutes on a treadmill at a speed of 25 m/min. After exercise, the mice given AST had far lower levels of plasma fatty acids than the animals on a regular diet. Exercise dramatically lowered intermuscular pH, whereas AST ingestion prevented this drop. AST‐fed animals showed considerably higher levels of PGC‐1α and its downstream proteins than mice fed a conventional diet. The consumption of AST caused an increase in PGC‐1α in skeletal muscle, which can trigger mitochondrial aerobic metabolism and speed up the use of fat. The idea that AST consumption will lessen the atrophy of muscles caused by immobility in rats was investigated in a study. Following 14 days of consumption of each experimental meal, a plaster cast was used to immobilize one leg's hindlimb muscles in the plantar flexion position. After immobilization for ten days, the contralateral plantaris and atrophic muscles were isolated and their protein levels of CuZn‐SOD and specific proteases were measured. This allowed for an analysis of the degree of muscular atrophy. The degree of muscular atrophy resulting from immobility was considerably lower in animals fed AST compared to those fed a placebo. Moreover, the immobilization‐induced rise in the expression of calpain, CuZn‐SOD, cathepsin L, and ubiquitin in the atrophied muscle was dramatically inhibited by AST supplementation (Shibaguchi et al. 2016).

Aoi et al. (2018) assessed the influences of the few forms of AST on sports endurance in mice. Four groups of 8‐week‐old ICR mice were created: a control group; an esterified form of AST derived from *Haematococcus pluvialis* ; a nonesterified form of AST isolated from Phaffia rhodozyma; and a nonesterified form of AST manufactured. The running duration to exhaustion was the longest and the AST concentrations in the tissue and plasma were considerably greater in the group that was given AST from Haematococcus than in the other groups. In the skeletal muscle, Haematococcus AST raised the levels of 5′‐AMP‐activated protein kinase. C57BL/6J mice were used in another investigation. They were fed either normal chow (NC) or NC with AST (NC+ ST) and either a high‐fat diet (HFD) or an HFD with AST for a month. The findings showed that HFD mice treated with AST had a better metabolic state, as seen by a substantial drop in serum total triglycerides, cholesterol, and blood glucose. As demonstrated by the hyperinsulinemic‐euglycemic clamp trial, AST‐treated HFD mice also exhibited increased glucose metabolism through improving glucose incorporation into peripheral target tissues, like the skeletal muscle, as opposed to inhibiting gluconeogenesis in the liver. Also, in the skeletal muscle of HFD animals, AST activated AMPK and raised coactivator expressions and transcriptional factors, generating mitochondrial remodeling that included enhanced free fatty acid metabolism and mitochondrial oxidative phosphorylation component (Nishida et al. 2020). Using a mouse model, a study looked at how AST affected adult hippocampal neurogenesis (AHN) and spatial memory. To determine the impact of AST on AHN, mice given diets enriched with AST (0%, 0.02%, 0.1%, and 0.5%) had their dose–response studied. The assessment of hippocampal‐dependent cognitive function was done in combination with the AHN findings. The outcomes showed that at 0.1% and 0.5% dosages, AST improved cell survival and proliferation. Only 0.5% AST elevated the number of newborn mature neurons and improved their spatial memory. Potential AHN‐associated molecules (Itga4, Prl, and Il4) that were activated by AST were identified using transcriptomic profiling. Through the use of Ingenuity Pathway Analysis, their downstream components were found to be favorably linked with the enhancements in spatial memory caused by AST (Yook et al. 2016).

## 7 The Impacts of AST on Cognitive Function

Several aspects of human cognitive function, including processing speed, working memory/short‐term memory, episodic memory, response inhibition, attention, and cognitive shifting, have been studied in connection with AST (Hayashi et al. 2018; Nouchi et al. 2020). Although AST has been proposed to be helpful, it is crucial to properly evaluate the study since the conclusions drawn from it may have ramifications for both improving cognitive function and treating cognitive impairment or neurodegeneration. Moreover, the consequences mentioned above may have a major impact on life expectancy and quality (Dumurgier and Sabia 2021; Knight et al. 2020). Given the known connections between oxidative stress and the circumstances of neurodegeneration, cognitive decline, cognitive aging, and cognitive longevity, this is particularly true with regard to cognition (Glade 2010). The idea that AST supplementation can enhance working memory is supported by information in the literature. In a study by Satoh et al. (2009), the mean percent accuracy on a working memory test rose from 90.46% to 96.30%. Another theory about AST and reaction time is also supported by the results of the research described. Following supplementation, there was a decrease in response times from baseline on tasks involving simple reaction, choice reaction, split attention, delayed recollection, and working memory. This suggests that the effects of AST on response times vary depending on the task and demographic. A study's findings showed that the natural antioxidant AST and low‐intensity exercise, or moderate exercise, both had positive impacts on hippocampus neurogenesis and memory performance. Leptin (LEP), which is produced and functions in the hippocampus, mediated the increase of hippocampal‐based plasticity and cognition by ME in combination with AST. When administered independently, ME and AST increased neurogenesis and spatial memory in wild‐type (WT) mice experiencing ME with or without an AST for 4 weeks. AST improved spatial memory mediated by AKT/STAT3 signaling and raised hippocampus LEP (h‐LEP) protein levels. Human neuroblastoma cell lines are directly affected by AST therapy, which increases cell survival and raises LEP expression. Chronic LEP infusion into the lateral ventricles of LEP‐deficient animals (*ob/ob*) restored the synergy (Yook et al. 2019). Aging‐related musculoskeletal illness can be a dangerous condition that can result in fractures and reduced motor function, leaving a person bedridden. Increasing muscular function by the consumption of functional meals is an efficient therapy technique for musculoskeletal diseases. Mice with muscle‐specific SOD2‐deficiency (muscle‐SOD2−/−) exhibit significant workout disruption accompanied by elevated levels of mitochondrial ROS, muscle injury, and mitochondrial malfunction. In the current investigation, 96 substances, including antioxidants, were given to muscle‐SOD2−/−mice and their effects on treadmill performance were evaluated. The dietary functional food components gossypin, β‐hydroxy‐β‐methylbutyrate calcium, taxifolin, genistein, kaempferol, fumaric acid, and AST were among those that were supplied to the muscle‐Sod2−/− mice. These compounds boosted the mice's forced running time. Furthermore, the antioxidants trolox, troglitazone, MnTE‐2‐PyP, and tempol markedly improved the running ability of muscle‐SOD2−/− mice. These findings showed that antioxidant‐rich functional meals may help with motor function. As a model of muscle exhaustion, muscle‐SOD2−/− mice were useful for the in vivo screening of practical compounds that enhanced muscle performance and exercise (Shibuya et al. 2022) (Table 2).

**TABLE 2:** The effects of astaxanthin on cognitive function.

Animal	Dosage	Duration	Primary outcome	Results	References
Mice	AST: 0.5% w/w	4 weeks	Cognitive function	Enhanced spatial memory along with rising hippocampal neurogenesis	Yook et al. (2019)
Muscle‐specific SOD2‐deficient mice	Not reported.	96 h	Motor function	Enhanced motor function	Shibuya et al. (2022)

## 8 Molecular Mechanisms and Synergistic Therapeutic Effects of AST and Exercise in Cancer

AST, a potent carotenoid primarily derived from microalgae, has garnered significant attention due to its powerful antioxidant, anti‐inflammatory, and anticancer properties. As a natural product, it is well‐known for its ability to modulate oxidative stress and inflammation, two key factors that play a central role in cancer initiation and progression. Recent studies have indicated that AST acts through multiple molecular mechanisms, including the inhibition of oxidative damage to DNA, modulation of apoptotic pathways, and regulation of various signaling pathways involved in cell proliferation and metastasis (Faraone et al. 2020). Specifically, AST has been shown to enhance the activity of antioxidant enzymes such as SOD and glutathione peroxidase, which reduce the accumulation of ROS in cells, thereby protecting against cancer‐induced oxidative stress (Fiedor and Burda 2014).

Exercise, as a nonpharmacological intervention, has also gained recognition for its beneficial effects in cancer prevention and treatment. Physical activity induces a wide range of molecular changes, such as the activation of antioxidant pathways, improvement in immune function, and modulation of metabolic processes, which together contribute to reduced cancer risk and enhanced therapeutic outcomes (Loprinzi et al. 2012). Notably, exercise has been linked to reduced inflammation, increased mitochondrial biogenesis, and improved vascular function, which are critical in the context of cancer biology. Moreover, exercise can influence tumor growth through systemic changes in cytokines, adipokines, and myokines, which affect both the tumor microenvironment and distant organs (Pedersen and Febbraio 2008).

The combination of AST supplementation with exercise represents a promising therapeutic strategy, offering potential synergies in the fight against cancer. Several preclinical and clinical studies suggest that the co‐administration of AST and exercise may amplify their individual effects, particularly in modulating cancer cell apoptosis, reducing tumor progression, and improving overall health outcomes. For instance, AST's ability to mitigate oxidative stress and inflammation could complement the beneficial effects of exercise‐induced molecular changes, such as increased mitochondrial function and improved immune surveillance (Oharomari et al. 2021). Together, these interventions may create an environment that inhibits tumor growth while enhancing the body's ability to fight cancer. In terms of clinical application, the integration of AST and exercise may offer a multifaceted approach to cancer management. As a natural antioxidant, AST has the potential to alleviate the side effects of conventional cancer therapies such as chemotherapy and radiation, whereas exercise contributes to improved quality of life, better physical functioning, and reduced risk of cancer recurrence. Further clinical trials are necessary to better understand the optimal dosages, duration, and mechanisms through which this combination might exert synergistic effects.

These studies offer valuable insights into the molecular mechanisms through which AST and exercise may exert anticancer effects; however, their methodological designs reveal both strengths and notable limitations. Many preclinical studies demonstrate clear mechanistic pathways, such as the activation of antioxidant enzymes (e.g., SOD, glutathione peroxidase), suppression of NF‐κB signaling, and enhanced apoptosis in cancer cells. These mechanistic insights are supported by molecular assays (e.g., Western blotting, qPCR), which strengthen the biological plausibility of AST's anticancer effects. Some studies do a commendable job exploring the interaction between AST and exercise, showing additive or synergistic effects on mitochondrial biogenesis, immune function, and anti‐inflammatory signaling. These findings support the theoretical rationale for combining lifestyle and nutritional interventions. The use of various cancer‐related models—including chemically induced tumor models and high‐fat diet‐induced inflammation models—adds diversity to the evidence base and suggests that the effects of AST are not limited to a single type of cancer pathology. Besides, there are also some weaknesses for these studies. Despite promising preclinical findings, no randomized controlled trials (RCTs) in humans are presented to validate the synergistic use of AST and exercise in cancer patients. This significantly limits translational value. Most evidence comes from murine models, which may not accurately reflect human tumor microenvironments, immune responses, or pharmacokinetics. Moreover, dosing in animal studies (based on body weight) often does not translate linearly to human‐safe dosages. Although the studies discuss AST and exercise individually in cancer contexts, very few, if any, evaluate exercise regimens in animals with active tumors alongside AST supplementation. This is critical, as exercise can have complex effects on tumor vasculature and metastasis depending on intensity, timing, and tumor type. Many preclinical experiments assess short‐term outcomes such as tumor volume or enzyme levels without examining long‐term survival, recurrence, or metastasis, which are essential to determine true clinical relevance.

## 9 Molecular Mechanisms and Synergistic Therapeutic Effects of AST and Exercise in Cardiovascular Diseases

Cardiovascular diseases (CVDs), including hypertension, atherosclerosis, and heart failure, remain leading causes of morbidity and mortality worldwide. The molecular pathophysiology of these diseases is characterized by oxidative stress, inflammation, endothelial dysfunction, and metabolic disturbances. Recent research has highlighted the potential of natural compounds like AST, a potent antioxidant carotenoid, in mitigating these processes. AST, derived from microalgae and certain marine organisms, has been shown to exhibit remarkable anti‐inflammatory and antioxidant properties, making it an attractive candidate for cardiovascular therapy (Fiedor and Burda 2014). Through its ability to neutralize ROS, AST reduces oxidative damage to lipids, proteins, and DNA, thus improving vascular function and protecting against endothelial dysfunction, a critical early event in CVD pathogenesis (Pashkow et al. 2008) (Figure 2).

Exercise, as a well‐established nonpharmacological intervention, exerts numerous cardiovascular benefits. Physical activity improves endothelial function, reduces inflammation, enhances lipid metabolism, and optimizes blood pressure regulation (Pedersen and Febbraio 2008). Exercise has been shown to induce positive changes in the expression of various genes and proteins involved in vascular health, such as endothelial nitric oxide synthase, which plays a central role in vasodilation and blood flow regulation (Rush et al. 2005). Additionally, exercise enhances mitochondrial function, reduces sympathetic nervous system activity, and improves cardiac output, all of which are critical for cardiovascular health.

The combination of AST supplementation and exercise holds great promise in improving cardiovascular outcomes. The synergistic effects of these two interventions are thought to arise from their complementary molecular actions. AST's antioxidant properties can enhance the benefits of exercise by protecting against exercise‐induced oxidative stress, which can otherwise lead to muscle damage and inflammation (Fiedor and Burda 2014). Furthermore, AST may improve the anti‐inflammatory effects of exercise by modulating inflammatory cytokines, such as TNF‐α and interleukin‐6 (IL‐6), which are elevated in CVD (Oharomari et al. 2021). A research evaluated how four weeks of AST supplementation affected cyclists' antioxidant capacity and the production of cardiac troponins during exercise. Prior to and following four weeks of AST (20 mg/day) or placebo treatment, thirty‐two male cyclists with high levels of training were repeatedly put through a laboratory‐based standardized exercise program. The findings showed that, just after exercise, the presupplementation cycling trial significantly increased median cardiac troponin T concentrations. The mean baseline plasma AST concentrations rose considerably after four weeks of AST administration. However, exercise‐induced cardiac troponin T release was unaffected by daily AST intake. Moreover, alterations in antioxidant capacity indicators did not correspond with the rise in basal plasma AST concentrations. AST supplementation had no effect on either exercise‐induced creatine kinase or high‐sensitivity C‐reactive protein (Klinkenberg et al. 2013). Another study evaluated how AST supplements affected the oxidative damage that intense exercise caused to the heart and gastrocnemius muscles in mice. Three groups of C57BL/6 mice were created: one for vigorous activity, one for rest, and one for exercise combined with AST administration. Both exercise groups ran on a treadmill at 28 m/min until they were exhausted following three weeks of exercise acclimation. In the AST group, exercise‐induced increases in 4‐hydroxy‐2‐nonenal‐modified protein and 8‐hydroxy‐2′‐deoxyguanosinein the heart and gastrocnemius were attenuated. AST also reduced increases in myeloperoxidase activity in the heart and gastrocnemius, as well as increases in plasma creatine kinase activity. After three weeks of treatment, AST was shown to accumulate in the heart and gastrocnemius. In mice, AST can reduce the damage that exercise causes to the heart and skeletal muscle, including the related neutrophil infiltration that causes additional harm (Aoi et al. 2003). The study's objective was to find out how 12 weeks of high‐intensity exercise combined with AST supplementation affected the lipid profiles, insulin resistance, and adipokine levels of obese men. Four groups of seventeen participants each—supplement group (SG), control group (CG), training group (TG), and training plus supplement group (TSG)—were randomly selected among 68 obese guys. All metrics showed substantial disparities between the groups, according to the statistics. Furthermore, the levels of GDF8, CTRP2, and CTRP9 differed from CG. GDF15 levels decreased in both training groups, but GDF8 levels were comparable in the SG and TG groups. Exercise training and AST supplementation improved lipid and metabolic profiles while lowering body composition, BMI, and adipokines (Saeidi et al. 2023). Another research compared baseline and 3‐month AST supplementation to examine improvements in physical activity and health‐related quality of life based on summary scores for the physical and mental components of the Short Form‐8. 17 heart failure patients' data were evaluated. The Specific Activity Scale score as well as the physical and mental component summary scores improved after three months of using AST supplements. The % change in the Specific Activity Scale score was linearly correlated with the baseline heart rate or mental component summary score. Additionally, the percentage change in the physical component summary score was directly correlated with the ischemia etiology. Lastly, the percent change in the mental component summary score and the Specific Activity Scale score showed a linear association (Ishiwata et al. 2021). The combination may also improve endothelial function more effectively than either intervention alone, as both AST and exercise contribute to increased nitric oxide production and reduced vascular stiffness. The combined approach may offer a multi‐faceted strategy to tackle cardiovascular risk factors, particularly in individuals with metabolic syndrome or those at high risk for heart disease. Although the molecular mechanisms underlying the synergistic effects of AST and exercise are becoming clearer, clinical studies are needed to confirm their therapeutic potential in humans. Clinical trials investigating the combined effects of AST and exercise on cardiovascular health, especially in patients with hypertension, atherosclerosis, or heart failure, would provide invaluable insights into the optimal dosage, duration, and treatment protocols. Given the growing interest in natural products and lifestyle interventions, the combination of AST and exercise could offer an accessible and effective approach for the prevention and management of CVD.

Collectively, several studies cited in this section measured clinically meaningful cardiovascular biomarkers, such as total cholesterol, triglycerides, glucose, CK, cardiac troponins, and inflammatory cytokines (e.g., TNF‐α, IL‐6). The inclusion of these markers strengthens the link between AST, exercise, and potential cardiovascular benefits. Both animal and human studies incorporated controlled interventions (e.g., AST supplementation with and without exercise), allowing for comparisons between intervention and control groups. This supports the evaluation of additive or synergistic effects. Unlike in cancer models, a few studies here examined the effect of AST specifically in combination with exercise on cardiovascular function. For example, mouse treadmill protocols combined with AST administration assessed cardiac and skeletal muscle protection under stress, providing more integrated data. Some human trials explored the effect of AST on quality of life and activity tolerance in patients with heart failure, offering preliminary insights into clinical applicability. Although some studies showed improvement in oxidative stress markers and quality of life, others found no effect of AST on exercise‐induced cardiac biomarkers (e.g., cardiac troponin T). This inconsistency highlights a need for more standardized protocols and suggests that AST's effects may be context‐ or dosage‐dependent. Some trials, particularly those with smaller sample sizes, do not specify randomization or blinding, introducing potential bias. This undermines the strength of their conclusions. Human studies often included only healthy individuals or well‐trained athletes rather than high‐risk cardiovascular populations. As a result, the generalizability of these findings to patients with established CVDs is limited. Although studies used varying doses (e.g., 4–20 mg/day), few attempted to explore dose–response relationships, making it difficult to determine the optimal dose for cardiovascular benefits. Furthermore, safety at higher doses in populations with cardiovascular comorbidities is not well established. Although animal studies explored molecular signaling (e.g., AMPK activation, oxidative stress modulation), human studies typically reported clinical outcomes without assessing mechanistic biomarkers, leaving gaps in our understanding of how AST works in the cardiovascular system in vivo.

## 10 Molecular Mechanisms and Synergistic Therapeutic Effects of AST and Exercise in Diabetes

Diabetes mellitus, particularly type 2 diabetes mellitus (T2DM), is a chronic metabolic disorder characterized by insulin resistance, impaired glucose metabolism, and increased risk of cardiovascular complications. Over time, chronic hyperglycemia leads to oxidative stress, inflammation, and vascular damage, which are central to the pathophysiology of diabetes and its complications (Li et al. 2024). Effective management of T2DM often involves a combination of pharmacotherapy and lifestyle interventions, with a growing body of evidence supporting the therapeutic potential of natural compounds like AST, a potent antioxidant carotenoid, and exercise in managing the disease. AST has gained attention due to its strong antioxidant and anti‐inflammatory properties. It has been shown to reduce oxidative stress, which is a key factor in the development of insulin resistance and pancreatic β‐cell dysfunction in diabetes (Fiedor and Burda 2014). By neutralizing ROS and inhibiting the activation of inflammatory pathways, AST helps protect against the cellular damage caused by hyperglycemia and contributes to improved insulin sensitivity (Li et al. 2020). Moreover, AST has been demonstrated to modulate key molecular signaling pathways, including the NF‐κB pathway, which plays a central role in the inflammatory response and insulin resistance (Fang et al. 2023). AST's ability to regulate lipid metabolism also contributes to its therapeutic effects in diabetes. By decreasing the levels of triglycerides and enhancing the function of high‐density lipoprotein, it helps reduce the risk of cardiovascular complications associated with diabetes (Urakaze et al. 2021). Furthermore, studies suggest that AST may improve mitochondrial function and glucose uptake in muscle cells, thereby enhancing overall metabolic health (Nishida et al. 2020).

Exercise is widely recognized as one of the most effective nonpharmacological strategies for managing diabetes. Physical activity improves insulin sensitivity, reduces blood glucose levels, and enhances cardiovascular health (Sellami et al. 2025). The molecular mechanisms through which exercise exerts these beneficial effects include increased muscle glucose uptake via the insulin‐independent pathway, upregulation of glucose transporter type 4, and enhanced mitochondrial biogenesis (Rush et al. 2005). Additionally, exercise induces the release of myokines, which are signaling proteins produced by muscles that play a role in glucose homeostasis, inflammation, and fat metabolism (Brandt and Pedersen 2010). Exercise also reduces systemic inflammation, a key contributor to insulin resistance and the progression of diabetes. It has been shown to lower circulating levels of pro‐inflammatory cytokines such as TNF‐α and IL‐6, which are elevated in individuals with T2DM (Huo et al. 2024). Moreover, regular exercise helps regulate lipid profiles, decreases visceral fat, and improves endothelial function (Mendelson et al. 2015), all of which contribute to the prevention and management of diabetic complications.

The combination of AST supplementation and exercise holds significant promise in enhancing the therapeutic effects in diabetes management. Both interventions target key mechanisms underlying the disease, such as oxidative stress, inflammation, and insulin resistance, suggesting a complementary approach (Fiedor and Burda 2014). AST's potent antioxidant properties can help mitigate the oxidative stress induced by exercise, which can lead to muscle damage and inflammation if not properly managed. Additionally, AST may amplify the benefits of exercise on insulin sensitivity and glucose metabolism, potentially leading to improved outcomes for individuals with diabetes. Furthermore, the synergistic effects of AST and exercise could extend to the prevention of diabetic complications. By reducing inflammation and improving lipid metabolism, the combination may help prevent the development of cardiovascular disease, which is a common comorbidity in diabetes. Although promising, the clinical application of AST and exercise as a combined therapeutic strategy for diabetes requires further investigation. Clinical trials are necessary to determine the optimal dosage of AST, the duration of supplementation, and the most effective exercise regimens for maximizing therapeutic outcomes. Additionally, understanding the long‐term effects of this combination on glycemic control, metabolic health, and diabetes‐related complications will be essential for establishing its role in clinical practice. Future research should also explore the potential of AST and exercise as adjunct therapies in individuals who are unable to achieve adequate glycemic control with conventional treatments.

Despite referencing some promising clinical results, there is a general lack of well‐powered human trials assessing the combined effect of AST and exercise on diabetic populations. Most of the evidence remains preclinical, restricting the applicability to real‐world settings. Several studies fail to provide details regarding the type, frequency, or intensity of exercise regimens when used in combination with AST, making it difficult to replicate or compare findings. This omission weakens the interpretability of the synergy claim. Most studies do not differentiate between prediabetes, early‐stage type 2 diabetes, or advanced cases, nor do they stratify outcomes based on glycemic control or insulin dependence. As a result, it is unclear which patient populations might benefit most from AST supplementation. As with other sections, the durations of intervention are often short (typically under 12 weeks), which is insufficient to evaluate long‐term glycemic control, prevention of complications, or durability of insulin sensitivity improvements. Many patients with diabetes are on medications like metformin, insulin, or SGLT2 inhibitors. None of the referenced studies investigate potential interactions between AST supplementation and these treatments, which is a critical omission for clinical relevance. The studies vary widely in the doses of AST used, with no clear rationale or discussion regarding optimal dosing for glycemic control. In addition, safety profiles specific to diabetic populations—particularly regarding renal or hepatic function—are not addressed.

## 11 Molecular Mechanisms and Synergistic Therapeutic Effects of AST and Exercise on Liver Function

Liver dysfunction, encompassing a wide array of conditions such as fatty liver disease, hepatitis, cirrhosis, and liver cancer, remains a significant global health concern (Clark et al. 2002). The liver plays a critical role in detoxification, metabolism, and storage, making its proper functioning essential for maintaining overall health (Clark et al. 2002; Brunt et al. 2015). However, lifestyle factors such as poor diet, lack of exercise, and environmental toxins can impair liver function, leading to chronic liver diseases and, in severe cases, liver failure or hepatocellular carcinoma. Therefore, finding effective therapeutic strategies for improving liver health is of paramount importance. Despite these promising findings, the precise molecular mechanisms underlying the synergistic effects of AST and exercise on liver function remain incompletely understood (Brunt et al. 2015). The purpose of one study was to investigate how AST supplementation affected the oxidative damage to the rats' livers caused by acute high‐intensity exercise (Zhang et al. 2022). Acute high‐intensity exercise may cause liver damage, according to the study's indicators assessment results. However, AST intervention will significantly reduce MDA concentration, lower ALT levels, and increase SOD activities and p‐GSK‐3β expression to help rats recover from exercise‐induced injuries. Additionally, by raising AMPKα1 phosphorylation and activating the transcription factor Nrf2, AST also upregulates the expression of the AMPK/Nrf2 signal pathway. This could improve antioxidant capacity and repair oxidative damage caused by exercise by enhancing the transcriptional translation of the downstream HO‐1 protein (Zhang et al. 2022). The impact of AST on muscle lipid metabolism during exercise was examined in a different investigation. ICR mice were split into four groups: running exercise, AST‐treated exercise, sedentary, and sedentary treated with AST. Exercise groups ran on treadmills following a 4‐week course of therapy. Compared to mice on a regular diet, AST enhanced fat utilization during exercise by extending the running time until exhaustion. AST enhanced the colocalization of fatty acid translocase with carnitine palmitoyltransferase I (CPT I) in skeletal muscle. Additionally, it was shown that exercise enhanced the hexanoyl‐lysine modification of CPT I, whereas AST inhibited this rise (Aoi et al. 2008). The human body produces more ROS when engaging in strenuous physical exercise, as indicated by elevated levels of malondialdehyde (MDA). Strong antioxidants like AST and exercise can change MDA levels. Therefore, the goal of the study was to determine how AST affected the dynamic pattern of MDA in male trainees following intense physical exercise. With the exception of the untrained male group that received a placebo, the data demonstrated that MDA was comparable before the test, increased considerably after the test, began to decline at the sixth hour posttest, and returned to baseline at the twenty‐fourth hour. The group of untrained males receiving a placebo had the highest mean MDA, whereas the group of trained males receiving an AST pill had the lowest mean (Sylviana et al. 2017).

Collectively, the use of high‐intensity or exhaustive exercise protocols to induce liver stress models a realistic scenario where oxidative injury occurs, making these findings particularly applicable to athletes or individuals engaged in strenuous physical activity. The section makes a clear attempt to evaluate the combined effects of AST and exercise on liver outcomes, rather than looking at each intervention in isolation—an important step toward exploring their synergy. However, most studies assess AST's effects on healthy or exercise‐stressed animals rather than established models of liver disease (e.g., NAFLD, NASH, hepatitis). Thus, it is unclear whether AST could provide benefits in chronic liver pathologies, where inflammation and fibrosis are dominant. Although molecular and biochemical markers are well‐measured, there is a lack of assessment of functional outcomes (e.g., liver histopathology, fibrosis scoring, hepatic insulin sensitivity), which are crucial to validate therapeutic relevance. Furthermore, the exercise regimens used to induce hepatic stress vary significantly in terms of intensity, duration, and frequency. Without standardization, it's difficult to compare results across studies or understand how exercise may independently or synergistically influence liver outcomes with AST.

## 12 The Effect of AST and Exercise on Neurological Protection

Tests for AST intake have been conducted for a variety of disorders, including diabetes, cardiovascular disease, dementia, and cancer, where inflammation and oxidative damage are important factors in the pathophysiological course of the disease (Ambati et al. 2014). One category of disorders where inflammation and oxidative damage play a major developmental role in furthering cognitive loss is dementia (Raz et al. 2016). In a model of AD using humanized APP mice, AST was demonstrated to have preventative benefits (Grilo and Mantalaris 2019). After nine months, the AST group had fewer AD onsets than the control group (Hongo et al. 2020). Remarkably, AST was discovered in brain tissues in rat and monkey studies (Choi et al. 2011; Nakamura et al. 2020), particularly in the cortex and hippocampus. This finding raises the possibility that AST might pass across the blood–brain barrier and directly impact cognitive performance. It is now known that exercise can be used to cure or prevent dementia without the use of pharmaceuticals (Ahlskog et al. 2011). Numerous molecular mechanisms, such as Irisin‐myokine release (Lourenco et al. 2019), neurotrophic factors such as brain‐derived neurotrophic factor (BDNF) (Choi et al. 2018) or insulin‐like growth factor 1 (IGF‐1) (Titus et al. 2021), dopamine turnover modulation (Koizumi et al. 2021), or improved cardiovascular structure (Samieri et al. 2018), can account for these neuronal benefits of sports on brain role. The well‐established advantages of sports on cognitive performance, however, are not well understood in relation to how dietary nutrients may enhance them. Remarkably, an animal study indicates that AST consumption enhances exercise‐induced neurogenesis even more (Yook et al. 2019).

Although opinions on how new neurons affect cognitive function are still divided, it is generally agreed that this process plays a part in neuroplasticity, learning, and memory (Polotow et al. 2014). Additionally, this same data implies that brain‐derived leptin signaling mediates the combined effects of AST and exercise on promoting neurogenesis (Yook et al. 2019). Increased hippocampal connections and plasticity are the major biochemical mechanisms by which leptin affects learning and memory (McGregor and Harvey 2018). The impacts of sports training on leptin levels were already well documented, whereas additional research is required to verify the impacts of AST on leptin metabolism (Fedewa et al. 2018). Enhancing cognitive performance, thus, may be a fruitful area of study to assess the complementary or synergistic benefits of AST and exercise. Studies conducted in vivo and in vitro have demonstrated the beneficial influences of AST on neuronal apoptosis (Wu et al. 2015; Bahbah et al. 2021). Simple definitions of neuronal apoptosis include “cell death” and the turnover of cells, and the operation of the immune system (Elmore 2007). However, apoptosis is supposed to have a part in a number of human‐based cognitive illnesses when unfavorable and uncontrolled pathogenic circumstances exist (Shao et al. 2016). Oxidative damage underlying processes connect AST to neuronal apoptosis. Oxidative stress may result in the production of ROS and excitotoxicity as a result of following effects (Barnham et al. 2004). Thus, neuronal apoptosis might provide a basis for AST‐based supplement‐based therapies. The aforementioned endeavor is further supported by more study. It has been reported that the loss of the natural antioxidant enzymes like SOD and catalase is harmful to cognitive function. It's been suggested that AST mitigates the unfavorable consequences linked with it (Grimmig et al. 2017). This conclusion is crucial since it has been demonstrated that the aforementioned molecules and/or enzymes become less effective with age. As a result, improving them may have a major impact on age‐related neurodegenerative diseases as well as overall brain aging (Stranahan and Mattson 2012).

Taken together, several studies explore the molecular mechanisms by which AST may exert neuroprotective effects, including its interaction with the AKT/STAT3 signaling pathway, enhancement of BDNF, and reduction in oxidative stress and inflammation. These mechanistic insights are crucial for understanding the biochemical rationale behind AST's potential cognitive benefits. Animal studies showing AST accumulation in brain regions (e.g., cortex, hippocampus) provide essential evidence that AST can cross the BBB—a key prerequisite for any neuroprotective agent. The synergistic effects of AST and ME on hippocampal neurogenesis and memory performance are a valuable contribution. These studies support the idea that AST may amplify the cognitive benefits of exercise, potentially through modulation of leptin signaling and neurotrophic pathways. Although some rodent studies show improvements in spatial memory and neurogenesis, not all of them corroborate these findings with histological or molecular confirmation (e.g., neuron counts, synaptic plasticity markers), limiting interpretability. Several studies report beneficial effects of AST at specific doses (e.g., 0.1%–0.5% in the diet), but there is no systematic evaluation of dose–response relationships, optimal timing of intervention, or potential toxicity at higher concentrations. Many neurological disorders, especially Alzheimer's disease and age‐related cognitive decline, display sex‐ and age‐dependent differences. Yet, the studies reviewed do not stratify their results based on these variables, which could mask important findings. In studies combining AST with exercise, the specific contribution of each intervention to the observed outcomes is not always clearly delineated. Without factorial design (e.g., AST only, exercise only, combined), it is hard to attribute results to synergistic effects rather than one dominant factor.

## 13 The Influences of AST on Sports Performance and Molecular Signaling in Human Studies

**FIGURE 3:**
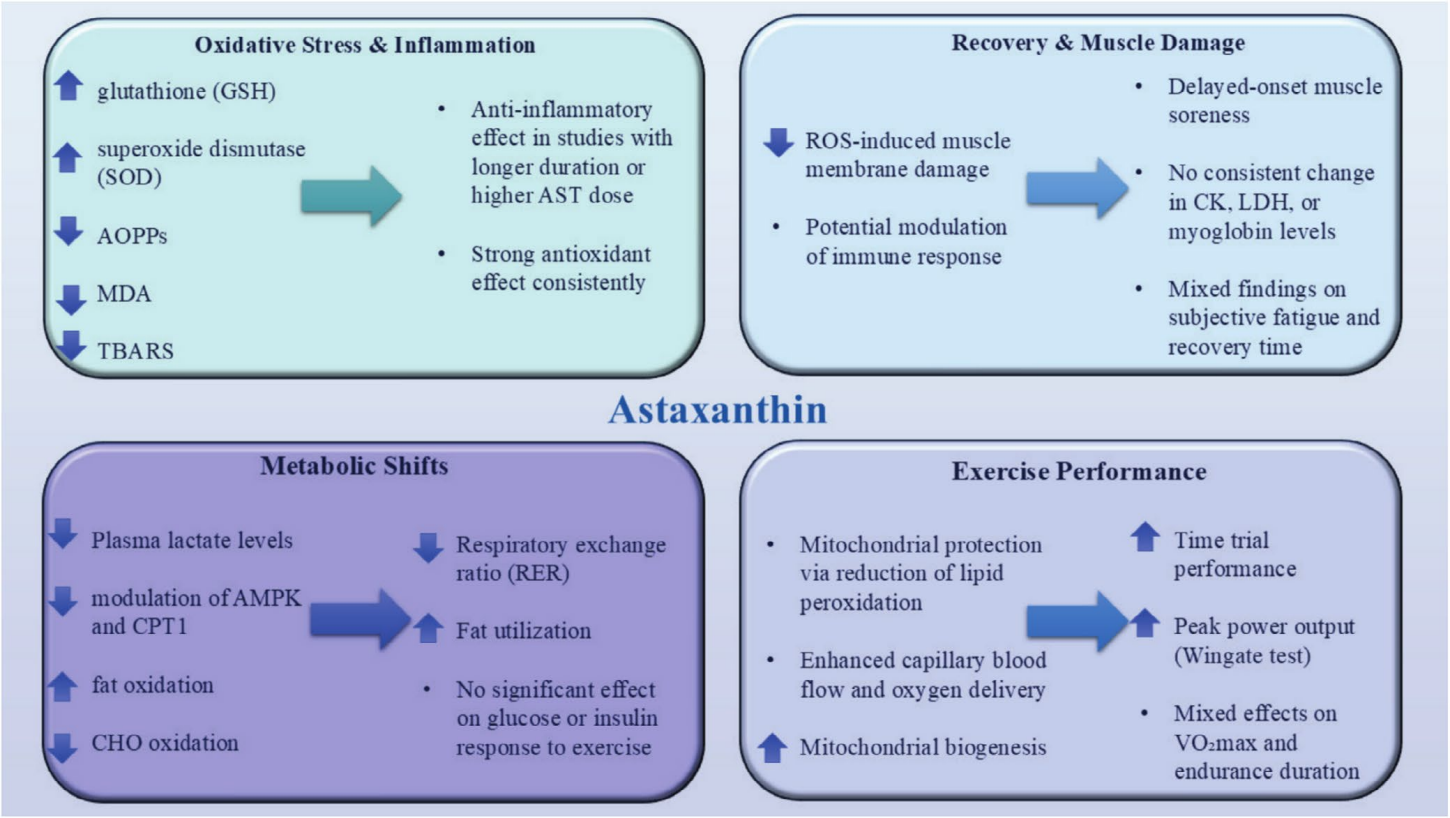
Mechanistic summary of astaxanthin's (AST) effects on exercise performance, metabolism, oxidative stress, and recovery in human trials. AST, a marine xanthophyll carotenoid, has demonstrated multifaceted benefits in exercise contexts. It enhances performance potentially through mitochondrial protection, improved oxygen transport, and vascular effects. Metabolic changes, such as increased fat oxidation and reduced lactate accumulation, are inconsistently observed but may relate to AMPK and CPT1 modulation. AST consistently reduces oxidative stress by scavenging reactive oxygen species and boosting endogenous antioxidants like glutathione and SOD. Anti‐inflammatory effects, likely mediated through NF‐κB inhibition, are evident in some long‐duration studies. Although AST may reduce subjective muscle soreness, evidence for its effect on biochemical markers of muscle damage remains mixed. Variability in findings across studies may stem from differences in dosage, duration, training status of participants, and exercise protocols. Overall, AST shows promise as a nutraceutical to support exercise performance and recovery, warranting further mechanistic and clinical exploration.

Among papers on human studies, there are 21 studies related to the objective of the current review (Table 3 and Figure 3). It is important to highlight that all of the AST utilized in human studies described here came from natural bases, not artificial ones. However, at this time, conclusions on the ergogenic influences of AST in humans cannot be drawn because of the small number of studies. Animal research has, predictably, produced more encouraging findings. This is typical of translation studies as using animals in research reduces the amount of potential confounding variables. The degree of fitness attained by research participants might be another reason. In all investigations, nonsedentary individuals undergoing advanced protocol training were examined for AST supplementation. Thus, it would be appropriate to evaluate AST supplementation in sedentary participants following low‐ to moderate‐intensity exercise programs. In one study, 21 elite cyclists were randomly divided to intake either a placebo or 28 days of encapsulated AST (4 mg/day) supplementation to assess the impact of AST on cycling time trial (TT) performance and substrate metabolism. A VO_2_ max test was part of the testing, and on a different day, a 20‐km time trial was performed five minutes after a two‐hour continuous intensity preexhaustion ride that began at a 5% VO_2_ max following a 10‐h fast. Overall, the findings demonstrated that the AST group's 20 km TT performance had significantly improved. Power production was substantially higher (20 W) in the AST group (Earnest et al. 2011a). In another study, the effects of 12 mg/day AST supplements for 7 days on sports performance and metabolism over a 40‐km cycling time trial were examined. Twelve male riders with leisure cycling experience were enlisted. On the 7th day of supplementation, subjects rode a 40‐km time trial on a cycle ergometer while having their exercise metabolic indices monitored continuously. The results showed that after taking AST supplements, the time needed to finish the 40‐km cycling time trial was reduced by 1.2%, from 70.76 min in the placebo condition to 69.90 min in the AST condition. Also, whole‐body fat oxidation rates were higher, and the respiratory exchange ratio lower between 39 and 40 km in the AST condition (Brown et al. 2021).

**TABLE 3:** The effects of astaxanthin on sport performance and molecular signaling in human studies.

Participants	Dosage	Duration	Primary outcome	Results	References
Competitive cyclists	4 mg/day	28 days	Cycling time trial	Improvement in 20 km TT performance and power output	Earnest et al. (2011a)
Trained male cyclists	12 mg/day	7 days	Cycling time trial	Improvement in 40 km TT performance Increased whole‐body fat oxidation rates Reduced respiratory exchange ratio	Brown et al. (2021)
Subjects underwent exercise on power bike		28 days	Antioxidant capacity	Increased the antioxidant capacity Reduced lactate value	Wu, Sun, Chen et al. (2019)
Healthy men	Not reported	10 weeks	Resistance training‐induced adaptation	Increased maximal voluntary contraction and resting oxygen consumption	Kawamura et al. (2021)
Male soccer players	4 mg/day	90 days	Salivary IgA	Increased sIgA levels Reduced prooxidant‐antioxidant balance, plasma muscle enzymes levels	Baralic et al. (2015)
Healthy paramedic students	4 mg/day	6 months	Strength endurance	Increased the average number of knees bendings	Malmsten and Lignell (2008)
Young, well‐trained male cyclists or triathletes	20 mg/day	4 weeks	Endurance performance	No effect on endurance performance	Res et al. (2013)
Recreational runners	12 mg/day	8 weeks	Cardiorespiratory function	No improvement in maximal oxygen uptake (running VO_2_ max) or maximal power output (cycling watts) A significant ~10% lower average heart rate at submaximal running intensities	Talbott et al. (2016)
Active young men	6 mg/day	4 weeks	Markers of oxidative stress	Increased ∼7% glutathione No effect on hydrogen peroxide, malondialdehyde, advanced oxidation protein, mean fat oxidation rates	McAllister et al. (2021)
Soccer players	4 mg/day	90 days	Oxidative stress status	Increase total SH groups content and improve PON1 activity	Baralic et al. (2013)
Resistance‐trained men	12 mg/day	4 weeks	Markers of muscle damage	Did not affect markers of muscle damage, inflammation, or DOMS after an EIMD protocol	Waldman et al. (2023)
Resistance‐trained men	12 mg/day	4 weeks	Markers of delayed onset muscle soreness	Increased recovery by reducing DOMS without detriment to performance	Barker et al. (2023)
Male and female runners	8 mg/day	Crossover design with two 4‐week	Immune‐related plasma proteins	Did not counter exercise‐induced increases in plasma cytokines and oxylipins but was linked to normalization of postexercise plasma levels of numerous immune‐related proteins including immunoglobulins within 24 h	Nieman et al. (2023)
Male students’	12 mg/day	28 days	Antioxidant Capacity	AST accelerated the recovery of antioxidant capacity, the clearance of blood lactate and delay the increase of blood uric acid in the body within 1 h after exercise	Wu, Sun, Zhao et al. (2019)
Resistance trained men	4 mg/day	3 weeks	Markers of skeletal muscle injury	Did not favorably affect indirect markers of skeletal muscle injury following eccentric loading	Bloomer et al. (2005)
Competitive amateur cyclists	4 mg/day	28 days	Cycling Time Trial Performance	Improved 20 km TT performance	Earnest et al. (2011b)
Young healthy male volunteers	4 mg/day	30 days	Aerobic exercise recovery	AST improved exercise recovery No benefit for AST over placebo in response to heat stress	Fleischmann et al. (2019)
Overweight individuals	12 mg/day	4 weeks	Fat oxidation rates and blood lactate concentrations	Reduced CHO oxidation and heart rate	Wika et al. (2023)
Adult males	12 mg/day	28 days	Human metabolism	Marked changes in blood metabolites involved in amino acid metabolism and lipid metabolism pathways	Guo et al. (2021)
Adult males	12 mg/day	28 days	Plasma metabolites	Accelerated metabolic recovery induced by physical exercise	Wang et al. (2021)
Males with obesity	20 mg/day	12 weeks	Adipokine levels	Decreased adipokines levels, body composition, anthropometrical factors (BMI) Improved lipid and metabolic profiles	Saeidi et al. (2023)

High‐intensity sports and AST supplements were found to have different effects on uric acid and lactic acid levels, antioxidant capacity, and both. After 28 days of training, sixteen participants were split into two groups at random: group B (control) and group A (experimental). All individuals exercised on a power bike four weeks later. The results showed that the two groups' antioxidative capacities fell immediately after exercise, whereas lactic acid levels dramatically rose. However, in comparison to group B, the antioxidant capacity and lactate levels of the A group were much greater (Wu, Sun, Chen et al. 2019). A further study evaluated the impact of diets high in anabolic nutrients on the adaptation of muscles caused by resistance exercise. Groups of 26 men in good health were divided into control and intervention groups. Every participant engaged in a ten‐week resistance training regimen twice a week. Foods high in resveratrol, β‐carotene, and AST were given to the intervention group. Data indicated that both groups' skeletal muscle mass was greater following training than it was prior to training. Following training, the maximal voluntary contraction increased in the intervention group but not substantially in the control group. Only in the intervention group did training result in a greater resting oxygen consumption. Serum carbonylated protein levels tended to be lesser in the intervention group only immediately following exercise than they were before exercise (Kawamura et al. 2021). The effects of AST supplementation were assessed in a study on differences in total/differential white cell counts, biochemical parameters, oxidative stress status in plasma, and salivary IgA (sIgA). 40 male soccer players with training were randomized to receive either AST or a placebo. The findings showed that following 90 days of AST supplementation, there was an elevation in sIgA levels at rest and a reduction in prooxidant‐antioxidant balance. Regular exercise combined with AST supplementation caused a considerable decrease in the values of muscle plasma enzymes. The inflammatory response in the AST‐taking patients was significantly blunted, as evidenced by the rise in hs‐CRP levels and neutrophil count that was only observed in the placebo group (Baralic et al. 2015).

In another study, the endeavor was created to determine the impact of AST supplements on physical performance. By using standardized workouts, three physical characteristics were tracked: strength/endurance, strength/explosivity, and fitness. Following a 6‐month term of supplementation, the average number of knee bendings (squats) rose for patients receiving AST by 27.05 and for subjects receiving a placebo by 9.0. After the trial, there had been no discernible differences between the groups in any of the other examined indicators (Malmsten and Lignell 2008). To evaluate the impact of AST intake for four weeks on substrate utilization and the ensuing time trial performance, thirty‐two young, fit male cyclists or triathletes were given either a placebo or 20 mg/day of ASTA. Subjects engaged in a time trial lasting around an hour following 60 min of exercise (50% *W*_max_) both before and after the supplementation period. The findings showed that neither malondialdehyde levels nor antioxidant capability were impacted by AST. During submaximal exercise, whole‐body fat oxidation rates did not vary over time or across groups. Neither group's time trial performance showed any improvement (Res et al. 2013).

A moderate dosage of natural AST (NAST) supplementation (12 mg/day for 8 weeks) was studied to see how it affected recreational runners' cardiorespiratory performance during both greater and lower‐intensity exercise. NAST or a placebo was given as supplements to 28 recreational runners. Subjects completed a maximal cycling test (watts on a cycle ergometer) and a maximum running test (VO_2_ max on a treadmill) both before and after the supplementation period. The study's findings showed that supplementing with NAST did not increase either maximum oxygen uptake (running VO_2_ max) or maximal power output (cycling watts). Participants in the NAST group had a noteworthy ~10% decrease in average heart rate at submaximal running intensities (Talbott et al. 2016). In addition, McAllister et al. (McAllister et al. 2021) active young men were given a graded exercise test to examine the impact of 6 mg/day AST supplements on substrate metabolism and indicators of oxidative stress. For four weeks, fourteen males received a placebo and an AST supplementation of six milligrams per day, with a one‐week washout interval in between. A fasting blood sample was taken at the end of each supplementation session to evaluate glutathione, advanced oxidation protein products (AOPPs), MDA, and hydrogen peroxide levels. Following each treatment, participants underwent a graded exercise test to ascertain substrate use during activity at escalating intensities. After AST, glutathione was approximately 7% greater than with a placebo. MDA and plasma hydrogen peroxide did not vary across treatments. The reduction in AOPPs was around 28%, however not statistically significant. Mean rates of fat oxidation did not differ between treatments throughout the graded activity test; however, in both circumstances, there was a decline in fat oxidation from 50 to 120 W and from 85 to 120 W. Active young men who took a 6 mg/day AST supplements for 4 weeks saw a rise in glutathione levels; however, this supplements did not affect substrate consumption or indicators of oxidative damage during exercise.

A study looked at how AST affected soccer players' levels of oxidative stress and paraoxonase (PON1) activity. A total of forty soccer players were randomized to receive either AST or a placebo. Before, 45 days after, and 90 days after supplementation, blood samples were taken. Diazoxon and paraoxon were the two substrates used to measure PON1 activity. The following oxidative stress indicators were also investigated: total sulphydryl group content (‐SH groups), redox balance, AOPPs, and thiobarbituric acid‐reactive substances (TBARS). Supplementation and training were found to have a substantial interaction impact on PON1 activity toward paraoxon. After ninety days, the AST group showed a rise in PON1 activity toward diazoxon, but the placebo group did not affect any discernible change. Only in the AST group did SH group content increase from the pre‐ to postsupplementation period. Following 45 days and 90 days of consistent soccer training, respectively, TBARS levels rose in both groups. All treatment groups saw a substantial drop in redox balance in response to frequent training (Baralic et al. 2013). Furthermore, exercise‐induced muscle damage (EIMD) has a well‐documented history of reducing exercise performance due to increased inflammation and subjective pain. It has been suggested that AST, with its strong antioxidative qualities, might be a useful dietary supplement to support decreasing delayed‐onset muscle soreness (DOMS) and improve performance and recovery. Waldman et al. (2023) were assessed on the influences of AST on DOMS, indicators of inflammation, substrate metabolism, and anaerobic function. Following each supplementation period, participants finished two trials: trial 1 involved a 30‐s Wingate and a graded exercise test (GXT), and trial 2 involved an EIMD protocol followed by the pre‐ and postcollection of fasting blood samples (pre‐post) to measure cortisol, creatine kinase, insulin, C‐reactive protein, AOPPs, and IL‐6. There were no significant differences seen in the indicators of substrate metabolism during the GXT, Wingate variables, or markers of inflammation, DOMS, or muscle injury between the two groups. 4 weeks of AST supplements did significantly decrease oxygen consumption during the last stage of the GXT, as well as baseline insulin levels and systolic blood pressure.

In another research, the impact of 12 mg/day AST supplements for 4 weeks on subjective indicators of DOMS, performance, and recovery following an eccentric exercise session that damages muscle was examined. The subjects underwent 4 trials: trials One and Three used a leg press one repetition maximum test (1RM) followed by 5 sets of 10 repetitions at 65% of 1RM to cause muscle injury. 48 h later, trials 2 and 4 were performance trials with repetitions to failure at 65%, 70%, and 75% of 1RM. For trials One and Three, subjective indicators of DOMS and recovery were gathered at various posttrial time points. AST dramatically reduced subjective indicators of DOMS, without affecting performance (Barker et al. 2023). Using a multi‐omics approach, a study looked at how well AST consumption over four weeks might moderate immunological dysfunction and inflammation brought on by exercise. After 4 weeks of supplementation, study participants were assigned to either AST or placebo groups. They then ran 2.25 h at about 70% VO_2_ max, which included 30 min of 10% downhill running. Algal AST weighed 8 mg in each AST pill. Six blood samples were collected at 1.5, 3, and 24 h after exercise, as well as before and after supplementation (an overnight fast). The study's findings showed that a 2.25‐h running session significantly increased inflammation, pain, and muscle injury. Muscular injury, exercise‐induced muscle soreness, and elevations in six plasma cytokines and 42 oxylipins were not affected by AST supplementation. Notably, throughout the 24‐h recovery period, AST supplementation reversed the declines in 82 plasma proteins brought on by exercise. The majority of these proteins were shown to be engaged in immune‐related processes, including humoral immune system responses, complement activation, and defensive responses, according to biological process analysis. There were found to be twenty plasma immunoglobulins that varied substantially between the AST and placebo studies. In the AST, but not in the placebo trial, IgM levels dramatically dropped later exercise but then rebounded following the 24‐h postexercise recovery period (Nieman et al. 2023).

To investigate the influences of AST supplementation on the body's ability to metabolize uric acid, blood lactic acid, and antioxidants throughout the hour‐long recovery phase following acute high‐intensity exercise. Randomly chosen, sixteen male students were split into two groups: group B (control) and group A (experimental). Group B received a placebo for 28 days, whereas Group A received AST at a moderate dosage of 12 mg/day. After four weeks, each participant worked out on a power cycle. The results demonstrated that: (1) The antioxidant capacity of groups A and B rose consistently one hour after exercise, with group A value exceeding group B. Lactic acid levels in both groups was significantly reduced and the levels of group A was lower than B. Uric acid levels increased in both groups and the level of A was lower than B (Wu, Sun, Zhao et al. 2019). In another study conducted by Bloomer et al. (2005), the influences of AST on markers of skeletal muscle damage were evaluated. Twenty males with resistance training were randomized to either AST or placebo. The subjects took the prescribed medication for 3 weeks leading up to the eccentric exercise (10 sets of ten repetitions at 85% of the maximal number of repetitions) and for 96 h following the exercise. Measurements were made of muscular soreness, creatine kinase, and muscle function both before and up to 96 h after exercise. All dependent variables showed a comparable response for both treatment groups, suggesting that AST supplementation had no beneficial effect on indirect indicators of skeletal muscle damage following eccentric loading in males who had undergone resistance training. In a study, the impact of AST intake on lipid metabolism and cycling time trial (TT) performance was evaluated. Following a 10 h fast, subjects allocated 21 competitive amateur cyclists to 2 h of constant‐load riding at 5% below the power output linked to 4 mmol/L of lactic acid buildup as assessed by a VO_2_ max test. Next came a 20‐km time trial for each rider. Participants either got a placebo or AST (4 mg/day) for 28 days. The results showed that the AST group, but not the placebo group, had notable gains in 20 km time trial performance. Comparisons between the groups indicated a substantial change between the AST and placebo groups. Moreover, the AST group's power production improved dramatically, whilst the placebo group showed no change (Earnest et al. 2011b).

An investigation was conducted on the impact of xanthophyll carotenoid AST supplementation on exertional heat strain and human physical performance. 22 male volunteers were given either AST or a placebo for thirty days. They underwent heat tolerance testing (HTT) and VO_2_ max test both before and after the supplementation. During the VO_2_ max test, heart rate (HR), blood lactate, relative perceived effort, and gas exchange were assessed. HTT recorded the following parameters: skin temperature, rectal temperature, heart rate (HR), sweat rate, and RPE. Before and after the test, serum heat shock protein 72, CRP, creatine phosphokinase, and lipid profiles were examined. The results indicated that both the decrease in oxygen uptake after recovery and the rise in lactate levels produced by the VO_2_ max test were considerably reduced in the AST group. There were no discernible variations in the VO_2_ max or anaerobic threshold. There were no discernible physiological or metabolic alterations in the HTT (Fleischmann et al. 2019). In another trial, nineteen volunteers were enrolled and given a placebo or 12 mg of AST every four weeks as a supplement. On a cycling ergometer, subjects finished a graded exercise test to look at variations in substrate oxidation rates. To investigate changes in lactate and glucose levels, carbohydrate (CHO) and fat oxidation rates, rating of perceived exertion (RPE) and heart rate, 5 stages were performed, each lasting 5 min and increasing resistance by 15 W per stage. Although rates of fat oxidation, glucose and lactate levels, or RPE did not alter, CHO oxidation in the AST group alone significantly decreased after supplementation. Moreover, during the graded exercise test, the AST group's heart rate decreased by 7% (Wika et al. 2023).

The impact of the naturally occurring antioxidant AST on the human body's functioning was investigated from the perspectives of metabolic network regulation and metabolites. Sixteen adult males were chosen, and they were split into two groups—a control group and an experimental group—equally and at random. All of the individuals had their fingertip blood drawn, whereas they were fasting to measure their blood lactate levels and antioxidant capacity. The β‐hydroxybutyric acid and differential metabolites (DMs) acetoacetic acid were involved in the ketone body anabolism and catabolism pathways; glutamic acid, alanine, and glutamine were involved in the alanine, glutamic acid, and aspartic acid metabolism pathways; and betaine (Bet), DMs creatine, and glycine were involved in the g serine, lysine, and threonine metabolism pathways. The experimental group had a considerably increased antioxidant capacity, although lactate levels showed the opposite pattern. Three possible DMs were identified immediately following exercise, and only aspartate, glutamine‐related alanine, and glutamic acid metabolic pathways were shown to have a significant impact on them. Antioxidant capacity declined following exercise in both groups, but it was meaningfully higher in the experimental group compared with the control group. Following exercise, lactate levels meaningfully increased in the experimental group and decreased meaningfully in the control group (Guo et al. 2021).

In another research, sixteen adult male subjects were split into two groups at random: the experimental group M (supplement medium dosage AST: 12 mg/day) and the control group C. Subjects engaged in acute activity by loading a weight of 0.075 kg/kg and pedaling a bicycle at full strength for intervals of 30 s to 3 min. 34 types of metabolites were tested from the two groups, and the results showed that the participants' metabolites varied at different time points. Three metabolites showed variations between the two groups one hour postexercise: glycerol, creatine, and β‐hydroxybutyrate. Group M had much lower amounts of glycerol and β‐hydroxybutyric acid compared to group C, but significantly greater levels of creatine. Leucine (Leu), citric acid (CA), and valine (Val) were the metabolites that both groups shared, and their levels were significantly lower in comparison to the resting state one hour after exercise. Glycerol and methionine (Met) and were the two distinct metabolites that separated the two groups during the hour following exercise. Group M's glycerol levels were considerably lower than group C's, whereas group M's Met levels were significantly greater. The levels of glucose, creatine, and glycerol‐co‐metabolites were significantly elevated in the participants in groups C and M one day following exercise (Wang et al. 2021). The purpose of another study was to look at how 12 weeks of high‐intensity exercise with AST supplementation affected the insulin resistance, lipid profiles, and adipokine levels in male obese patients. 4 groups of seventeen participants each were randomly selected from a sample of sixty‐eight obese males: supplement group (SG), training group (TG), training plus supplement group (TSG), and control group (CG). For 12 weeks, the participants received either AST or a placebo (20 mg/day pill every day). The training regimen comprised three sessions per week and 36 sessions of high‐intensity functional training (HIFT), lasting 60 min each. For every indicator, there were notable variations between the groups. Post hoc analysis revealed that whereas GDF15 levels were comparable to CG, CTRP9, CTRP2, and GDF8 levels were not. GDF8 levels were comparable in the SG and TG groups, whereas both training groups had lower GDF15 levels (Saeidi et al. 2023).

## 14 Limitations and Therapeutic Avenues

### 14.1 Limitations

Although preclinical and animal studies suggest strong synergistic effects between AST and exercise, the number of large‐scale, human clinical trials remains limited. Most existing studies have small sample sizes, varying dosages of AST, and differing exercise regimens, making it difficult to generalize findings across broader populations. Longitudinal human studies are essential to confirm the therapeutic benefits, establish long‐term safety, and determine the ideal dosing and timing of AST supplementation in conjunction with exercise. Also, there may be individual variability in the effectiveness of AST and exercise due to factors such as genetics, baseline health conditions, and lifestyle behaviors. For example, genetic polymorphisms affecting drug metabolism or antioxidant responses could influence the bioavailability and efficacy of AST. Additionally, the response to exercise varies based on the type of exercise regimen, fitness levels, and preexisting conditions such as metabolic disorders or cancer stage. Although studies have shown positive effects of AST in various doses (ranging from 4 mg to 12 mg per day), there is no consensus on the most effective dosage for specific diseases, including cancer and metabolic conditions. The lack of standardized dosages and delivery methods, as well as the absence of comprehensive pharmacokinetic studies, limits the ability to apply AST as a mainstream therapeutic agent. The combined use of AST and exercise may interact with other pharmacological treatments. For instance, in cancer treatment, where patients are often prescribed chemotherapy and immunotherapies, there may be concerns regarding the interaction between antioxidants like AST and oxidative stress‐based cancer therapies. More research is needed to ensure that AST does not interfere with the therapeutic effects of existing treatments.

### 14.2 Therapeutic Avenues

Future research should focus on personalized medicine, tailoring AST supplementation and exercise protocols to individual patient characteristics, such as their genetic profile, disease stage, and overall health status. By identifying biomarkers of response, healthcare providers can better predict which patients will benefit most from this combination therapy, optimizing outcomes and minimizing side effects. In addition to exercise, combining AST with other natural compounds known for their synergistic effects (such as curcumin, resveratrol, or omega‐3 fatty acids) could further enhance therapeutic outcomes. These multi‐modal approaches may help address the complex nature of diseases like cancer and metabolic disorders by targeting various molecular pathways simultaneously, including inflammation, oxidative stress, and cellular apoptosis. Also, exercise regimens should be optimized for patients with cancer or metabolic diseases to maximize the therapeutic effects of AST. Incorporating a combination of aerobic, resistance, and flexibility training could provide a comprehensive approach to managing these diseases. Future clinical trials should assess the benefits of tailored exercise programs that complement AST supplementation to better understand how exercise modulates metabolic pathways and cancer progression. Further studies should delve into the molecular mechanisms underlying the synergy between AST and exercise, focusing on the roles of cellular pathways such as Nrf2 activation, PI3K/Akt/mTOR signaling, and autophagy. By understanding how these interventions interact at the cellular level, it will be possible to identify new therapeutic targets and refine treatment strategies. In addition, AST supplementation, combined with regular physical activity, could be considered as part of a holistic approach to disease prevention, particularly for high‐risk populations, such as individuals with a family history of cancer, obesity, or metabolic syndrome. Public health initiatives promoting lifestyle changes, including exercise and antioxidant‐rich diets, could be a cost‐effective and widely accessible strategy to reduce the burden of chronic diseases.

## 15 Future Prospective

Despite the promising evidence supporting the therapeutic potential of AST, several limitations within the current body of research must be acknowledged. First, a significant proportion of the evidence is derived from preclinical and animal studies, which, although mechanistically informative, may not fully translate to human physiology due to differences in metabolism, dosing, and study conditions. Second, the methodological quality of human studies varies widely, with inconsistencies in study design, sample size, supplementation duration, and participant characteristics, limiting the generalizability of findings. Moreover, although AST dosages ranging from 4 to 20 mg/day have been tested, there is no standardized protocol for dose optimization across specific health conditions, leaving clinicians and researchers without clear dosing guidelines. Another limitation is the lack of long‐term safety data, particularly at higher dosages or in populations with chronic diseases. Additionally, the potential interactions between AST and pharmacological treatments—especially in oncology or cardiovascular settings—remain underexplored. Finally, although the synergistic effects of AST and exercise appear beneficial, the type, intensity, and frequency of exercise that optimally complement AST supplementation have yet to be clearly defined.

Future research should prioritize large‐scale, placebo‐controlled clinical trials to evaluate the efficacy, safety, and optimal dosing of AST in diverse populations and disease contexts. Studies exploring its use in combination with specific exercise regimens and other dietary compounds (e.g., curcumin, resveratrol) may also provide insights into maximizing its therapeutic potential. Furthermore, investigating genetic or metabolic biomarkers that predict individual responsiveness to AST could pave the way for more personalized supplementation strategies. Collectively, addressing these gaps will enhance the clinical applicability of AST as a supportive intervention in both health promotion and disease management. Although human studies investigating AST supplementation have utilized a wide dosage range (4 mg to 20 mg/day), optimal dosing remains context‐dependent and insufficiently standardized. Lower doses (4–6 mg/day) have shown modest improvements in antioxidant status and exercise recovery, whereas higher doses (12–20 mg/day) are more commonly associated with improvements in endurance performance, inflammatory markers, and metabolic profiles. For instance, 12 mg/day for 8–12 weeks has been linked to enhanced fat oxidation and reduced exercise‐induced oxidative damage, whereas 20 mg/day has been explored in clinical settings involving obesity and metabolic disorders. However, no universal consensus exists regarding disease‐specific dosing, and further RCRs are needed to establish safe and effective dosing regimens tailored to various health conditions. Until such data are available, dosing decisions should be guided by the target outcome, individual health status, and duration of supplementation.

A critical evaluation of the included studies reveals notable variability in methodological rigor. Although most animal studies were well‐controlled and employed standard experimental protocols, many lacked blinding and sample size justification, which may introduce bias. Human studies also varied in quality, with differences in participant training status, intervention duration, and dosage of AST supplementation. Several trials lacked placebo control, randomization, or long‐term follow‐up, limiting the strength of their conclusions. Additionally, reporting on adherence, baseline nutritional status, and confounding variables was often insufficient. These methodological inconsistencies should be considered when interpreting the overall findings. Future research should adhere to standardized reporting guidelines such as CONSORT for human trials and ARRIVE for animal studies to ensure greater reproducibility, transparency, and comparability across studies.

## 16 Conclusions

The combination of AST supplementation and exercise presents a promising synergistic therapeutic approach for managing and preventing a range of diseases, including cancer and metabolic disorders. Both AST and exercise independently exert significant health benefits through their ability to reduce oxidative stress, modulate inflammation, and regulate key molecular pathways involved in cellular function and metabolism. When used together, these interventions may enhance each other's effects, creating a multifaceted strategy that targets various physiological mechanisms involved in disease progression and health maintenance. AST's potent antioxidant properties, along with its ability to modulate apoptotic and autophagic pathways, make it a powerful adjunct to exercise in the context of cancer and metabolic diseases. Exercise, in turn, promotes mitochondrial health, improves insulin sensitivity, and triggers beneficial adaptations in cardiovascular and immune function. Together, they may help optimize cellular function, reduce chronic inflammation, and protect against disease progression. Although the current body of evidence is promising, further clinical studies are necessary to better understand the optimal dosages, timing, and combinations of AST and exercise for different patient populations. Moreover, more research is needed to establish the long‐term safety and efficacy of this combined therapeutic approach in both diseased and healthy individuals. In conclusion, the synergistic effects of AST and exercise hold great potential for improving health outcomes across a variety of conditions, offering a natural and accessible therapeutic strategy with minimal side effects. Continued exploration of their combined mechanisms and clinical applications could pave the way for novel, integrative approaches to disease prevention and management, benefiting individuals with cancer, metabolic diseases, and those seeking to optimize their health.

## Author Contributions

**Wenwen Nie:** conceptualization (equal), data curation (equal), investigation (equal), methodology (equal), supervision (equal), validation (equal), visualization (equal), writing – original draft (equal), writing – review and editing (equal). **Jianmin Li:** conceptualization (equal), data curation (equal), investigation (equal), methodology (equal), validation (equal), visualization (equal), writing – original draft (equal), writing – review and editing (equal). **Sogand Rajabi:** conceptualization (equal), data curation (equal), investigation (equal), methodology (equal), supervision (equal), validation (equal), visualization (equal), writing – original draft (equal), writing – review and editing (equal).

## Ethics Statement

The authors have nothing to report.

## Consent

The authors have nothing to report.

## Conflicts of Interest

The authors declare no conflicts of interest.

## Data Availability

Data sharing not applicable to this article as no datasets were generated or analyzed during the current study.
